# Nodal status dictates divergent prognostic drivers in oral squamous cell carcinoma: metabolic burden in pN0 vs. sarcopenia and nodal burden in pN+

**DOI:** 10.3389/fonc.2026.1746241

**Published:** 2026-02-18

**Authors:** Shihui Shen, Wugang Zhou, Yuhua Hu, Ting Gu, Yubo Ma, Haihua Yuan, Feng Xu

**Affiliations:** 1 Department of General Dentistry, Shanghai Ninth People’s Hospital, Shanghai JiaoTong University School of Medicine, Shanghai, China; 2 Department of Emergency, Shanghai Ninth People’s Hospital, Shanghai JiaoTong University School of Medicine, Shanghai, China; 3 Department of Oral Pathology, Shanghai Ninth People’s Hospital, Shanghai JiaoTong University School of Medicine, Shanghai, China; 4 Department of Nuclear Medicine, Shanghai Ninth People’s Hospital, Shanghai JiaoTong University School of Medicine, Shanghai, China; 5 Department of Oncology, Shanghai Ninth People’s Hospital, Shanghai JiaoTong University School of Medicine, Shanghai, China

**Keywords:** L3-SMI, number of positive nodes, oral squamous cell carcinoma, survival analysis, Tumor-SUVmax

## Abstract

**Objectives:**

Oral squamous cell carcinoma (OSCC) exhibits heterogeneous outcomes based on nodal status, complicating personalized prognosis. This study aimed to identify nodal-specific prognostic factors in OSCC by integrating metabolic metrics from ^18^F-fluorodeoxyglucose Positron Emission Tomography/Computed Tomography, body composition (L3 skeletal muscle index, L3-SMI), and key pathological features, to refine risk stratification.

**Methods:**

We conducted a retrospective cohort study of 147 OSCC patients (74 pN0, 73 pN+) who underwent curative resection and neck dissection. Associations between metabolic metrics, L3-SMI, pathological factors, and overall (OS) or progression-free survival (PFS) were evaluated using Cox regression. Internal bootstrap validation (1000 repetitions) was performed to assess the stability and potential bias of the prognostic factors.

**Results:**

In pN0 patients, tumor maximum standardized uptake value (T-SUVmax) >13.27 emerged as an independent predictor of poor OS (HR = 10.24, *P* = 0.003) and PFS (HR = 8.23, *P* = 0.002), which was validated by bootstrapping. Among pN+ patients, ≥3 positive lymph nodes significantly predicted worse outcomes (OS HR = 4.15, *P* < 0.001; PFS HR = 1.97, *P* = 0.049), while higher L3-SMI served as a protective factor for survival (OS HR = 0.13, *P* = 0.047; PFS HR = 0.18, *P* = 0.024); both findings were supported by internal validation.

**Conclusions:**

We propose a nodal status-stratified approach for risk assessment in OSCC. For pN0 necks, high risk is characterized by elevated T-SUVmax, whereas in pN+ necks, high nodal burden and sarcopenia define increased risk.

## Introduction

Oral squamous cell carcinoma (OSCC) is the most common oral cavity malignancy, accounting for approximately 90% of cases. While multimodal treatment approaches have advanced, the 5-year survival rate remains suboptimal at 50-70%, highlighting an urgent need for more precise prognostic stratification (1, 2). Current prognostic models incorporate metabolic parameters from ^18^F-fluorodeoxyglucose Positron Emission Tomography/Computed Tomography (^18^F-FDG PET/CT) imaging, including tumor maximum standardized uptake value (T-SUVmax) (3, 4), tumor metabolic tumor volume (T-MTV) (5), and tumor total lesion glycolysis (T-TLG) (6), all of which demonstrate significant correlations with clinical outcomes. Nutritional status and body composition, often impacted by cancer-associated cachexia, are increasingly recognized as prognostic factors in malignancies such as pancreatic and lung cancer. In OSCC, the L3 skeletal muscle index (L3-SMI) has emerged as a novel prognostic indicator (7–9). However, most previous studies have analyzed combined pN0/pN+ patients, potentially obscuring critical nodal status-dependent prognostic differences. Recent work by Chang et al. (10) revealed distinct pathological prognosticators between pN0 and pN+ subgroups, with surgical margins, depth of invasion (DOI), and perineural invasion (PNI) being more predictive in pN0 cases, while extranodal extension (ENE) dominated in pN+ disease. However, a comprehensive, nodal-stratified analysis that integrates metabolic parameters, body composition, and detailed pathological features is still lacking. To address this gap, we conducted a comprehensive, nodal-stratified analysis to identify prognostic drivers by evaluating PET/CT-derived metabolic metrics, L3-SMI, and key pathological factors in OSCC.

## Methods

### Patients

We retrospectively analyzed consecutive OSCC patients treated between January 1, 2018, and June 1, 2024. Inclusion criteria were: preoperative ^18^F-FDG PET/CT within 4 weeks before surgery showing no distant metastases, curative resection of the primary tumor with neck dissection, pathological confirmation of OSCC, and ≥12 months of follow-up for surviving patients. Exclusion criteria included tumor recurrence, prior malignancy, treatment before PET/CT, blood glucose >200 mg/dL during PET/CT, or death within 30 days post-surgery. Patients were stratified by pathological lymph node status (pN0 vs. pN+).

### PET/CT acquisition and metabolic variables

Patients fasted for ≥6 hours before ^18^F-FDG injection. ^18^F-FDG was administered intravenously at 0.1 mCi/kg, PET/CT scans were conducted using the Ingenuity TF (Philips Medical Systems, Cleveland, USA) at Shanghai Ninth People’s Hospital (11). Images were acquired 60 minutes post-injection, skull base to mid-thigh/foot. Non-contrast CT (120 kVp; 150–200 mA; 3 mm thickness; 512×512 matrix) followed by PET (90 seconds/bed). PET data reconstructed on-scanner: OSEM, 3 iterations, 33 subsets. Tumor volumes of interest (VOIs) were auto-delineated via 40% SUVmax threshold (12), manually adjusted to exclude adjacent physiological ^18^F-FDG-avid structures. T-SUVmax, T-SUVmean, and T-MTV auto-calculated; T-TLG = T-SUVmean × T-MTV. In pN+ patients, the nodal maximum standardized uptake value (N-SUVmax) measured at most metabolically active lymph node; if no nodes exceeded background SUVmax, longest-diameter node on positive side selected.

### L3-SMI

Non-contrast CT images from PET/CT were analyzed using SliceMatic 5.0 software (Tomovision, Montreal, Canada). A cross-sectional CT image at the third lumbar (L3) level, clearly showing the L3 transverse processes, was selected. Paraspinal and parietal muscles were semi-automatically segmented using Hounsfield unit (HU) thresholds (-29 to +150). L3-SMI was calculated as cross-sectional muscle area (cm²) normalized to height² (m²). Chinese population-specific sarcopenia cutoffs were applied (men: <55.0 cm²/m²; women: <36.6 cm²/m²) (13).

### Pathological features

Primary tumor features (AJCC/UICC TNM staging, 8th edition) included tumor grade, PNI, lymphovascular invasion (LVI), surgical margin status, depth of invasion (DOI; categorized as ≤5 mm, >5–10 mm, or >10 mm), pathological T stage, and pathological N stage. For pN+ patients, lymph node characteristics evaluated included extranodal extension (ENE), lymph node yield (LNY; total nodes removed), number of positive nodes, and lymph node density [LND; ratio of positive to total nodes removed, also termed lymph node ratio (LNR)].

### Adjuvant treatment

Adjuvant therapy recommendations were determined by a multidisciplinary tumor board (MDT) involving oncologists, surgeons, radiologists, and nuclear medicine physicians, with treatment initiated within 6 weeks post-surgery. Radiotherapy (56–60 Gy total dose at 2.0 Gy per fraction via linear accelerator) and/or cisplatin-based chemotherapy were administered based on clinical status, pathological findings, and nodal involvement.

### Statistical analyses

Statistical analysis was performed using SPSS version 17.0, (Chicago, IL, USA) and MedCal19.6.1 (Ostend, Belgium). No missing data were identified in this study. Continuous variables were expressed as mean ± standard deviation or median (range), while categorical variables were presented as frequencies (percentages). Between-group comparisons were performed using Student’s t-test, Mann-Whitney U test, χ² test, or Fisher’s exact test as appropriate. Survival outcomes were analyzed using Cox proportional hazards regression models, with results expressed as hazard ratios (HRs) and 95% confidence intervals (CIs). Primary endpoints were progression-free survival (PFS) and overall survival (OS), defined from PET/CT date to OSCC-related recurrence/all-cause death (censored at June 1, 2025). Receiver operating characteristic curve analysis with Youden index identified optimal cutoffs for key variables (**Supplementary Table 1**), using OS as the endpoint. Univariate Cox models were followed by multivariate analysis (backward stepwise) with *P* < 0.05 variables. T-MTV was prioritized over T-TLG due to multicollinearity (VIF = 9.418). Kaplan-Meier curves and log-rank tests visualized survival differences. To evaluate the stability of the findings and quantify potential bias, internal validation of prognostic factors was conducted through bootstrap resampling with 1000 repetitions by using R version 4.1.0. A two-sided *P* < 0.05 was considered statistically significant.

## Results

### Overview of the pN0 and pN+ patients

A total of 147 OSCC patients were included, with demographic/clinical characteristics in **Table 1**. Median follow-up was 43 months (12–83): 42.5 months for pN0 and 44 months for pN+. During follow-up, 42 patients (29%) died, including 12 in the pN0 and 30 in the pN+ group. Disease progression or recurrence occurred in 52 patients (35%), with 15 in the pN0 and 37 in the pN+ group. Two patients developed second primary malignancies: one in the pN0 group (kidney carcinoma at 4 months) and one in the pN+ group (esophageal carcinoma at 36 months). Both were alive without OSCC recurrence at the end of follow-up. Adjuvant therapy was recommended for 39.2% of pN0 patients (29/74, all completed) and all pN+ patients (73/73), with 84.9% (62/73) completing it. Among pN+ patients who did not complete adjuvant therapy (9 due to physical status, 2 due to patient refusal), there were 7 males [L3-SMI: 35.71 (29.83–53.91)] and 4 females [L3-SMI: 35.71 (30.29–44.25)]; 81.8% (9/11) of these patients had sarcopenia.

**Table 1:** Demographic and clinical characteristics of the OSCC patients.

Characteristics	Total (n=147)	pN0 (n=74)	pN+ (n=73)
Age (median, range)	64(29-89)	67(31-86)	61(29-89)
Gender (Male *vs* Female)	110:37	53:21	57:16
Tumor location (Tongue/Cheek/Gingiva/Other)	73/27/21/26	33/12/17/12	40/15/4/14
Neck dissection level			
Affected side: I-II/I-III/I-IV/I-V	7/46/11/83	7/31/6/30	0/15/5/53
Contralateral side: I-II/I-III/I-IV/I-V	1/8/2/2	0/0/0/0	1/8/2/2
Total number of removed nodes (median, range)	23(3-75)	21(3-48)	30(8-75)
Number of positive nodes (median, range)		0	2(1-20)
pT stage* (T1/T2/T3/T4)	20/39/86/2	16/23/35/0	4/16/51/2
pN stage* (N0/N1/N2/N3)	74/22/29/22	74/0/0/0	0/22/29/22
TNM stage* (I/II/III/IV)	15/24/57/51	15/24/35/0	0/0/22/51
Adjuvant treatment			
None/Chemotherapy/Radiotherapy/CRT	52/2/60/33	45/2/25/2	6/0/35/32

OSCC, oral squamous cell carcinoma; CRT, chemoradiotherapy.

*AJCC/UICC TNM staging, 8^th^ edition.

A comparison of prognostic variables between pN0 and pN+ patients is presented in **Table 2**. Higher values of T-SUVmax, T-MTV, T-TLG, as well as younger age, were observed in pN+ patients compared to pN0 patients. Furthermore, DOI >5mm, PNI and poorly differentiated tumor grade were more frequently observed among pN+ patients.

**Table 2:** Comparison of potential prognostic variables in pN0 and pN+ patients.

Variable	Total (n=147)	pN0 (n=74)	pN+ (n=73)	*P* ^#^
Gender (Male *vs* Female)	110:37	53:21	57:16	0.475
Age (median, range)	64(29-89)	67(31-86)	61(29-89)	0.029*
Smoker (No *vs* Yes)	101:46	54:20	47:26	0.261
Drinker (No *vs* Yes)	116:31	63:11	53:20	0.063
T-SUVmax	13.15(3.02-38.42)	11.56(3.02-38.42)	14.41(3.70-31.74)	0.019*
T-MTV	5.76(0.64-79.04)	4.12(0.77-57.22)	7.74(0.64-79.04)	0.001**
T-TLG	44.52(2.87-1052.67)	27.71(3.32-457.73)	68.99(2.87-1052.67)	0.001**
L3-SMI (Male)	45.55 ± 8.46 (n=110)	46.14 ± 8.49 (n=53)	44.99 ± 8.47 (n=57)	0.479
L3-SMI (Female)	38.63 ± 5.94 (n=37)	38.98 ± 4.62 (n=21)	38.16 ± 7.47 (n=16)	0.686
pT Stage (T1–2 *vs* T3-4)	59:88	39:35	20:53	0.002**
DOI (mm) ≤ 5, >5-10, >10	28:35:84	20:21:33	8:14:51	0.006**
PNI (Negative *vs* Positive)	110:37	61:13	49:24	0.032*
LVI (Negative *vs* Positive)	145:2	74:0	71:2	0.245
Grade (Well, moderate, Poor)	8:130:9	8:66:0	0:64:9	<0.001***
Margin (Negative *vs* Positive)	141:6	71:3	70:3	0.986

^#^Comparison between patients with pN0 and pN+ neck, *P < 0.05, **P < 0.01, ***P < 0.001; T-SUVmax, tumor maximum standardized uptake value; T-MTV, tumor metabolic tumor volume; T-TLG, tumor total lesion glycolysis; L3-SMI, L3 skeletal muscle index; DOI, depth of invasion; PNI, perineural invasion; LVI, Lymphovascular invasion. Bold values indicate P < 0.05.

### pN0 cohort

**Figure 1:**
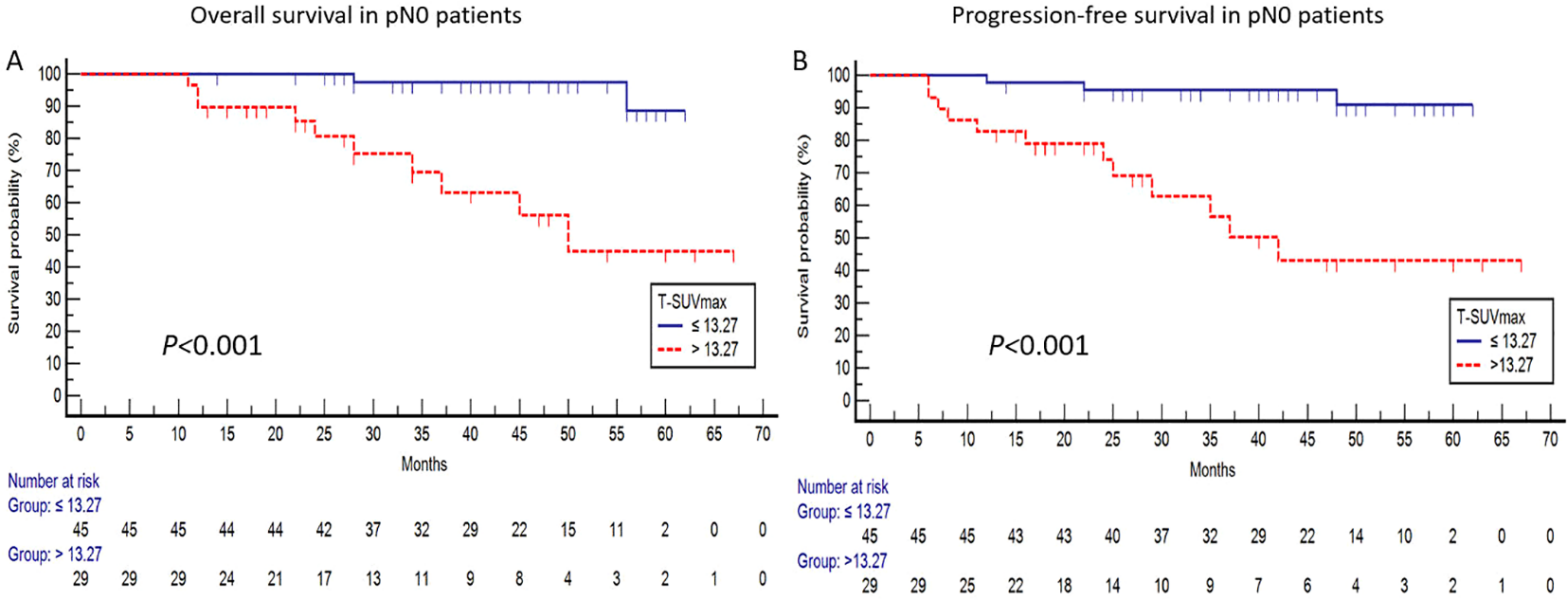
For pN0 patients, the survival curves using a T-SUVmax cutoff of 13.27 demonstrated significant differences in both OS [*P* < 0.001; **(A)**] and PFS [*P* < 0.001; **(B)**] between the high and low T-SUVmax subgroups.

In pN0 patients, T-SUVmax, along with T-MTV and T-TLG, emerged as significant prognostic factors for OS and PFS in the univariate analysis. In the multivariate analysis, T-SUVmax remained an independent predictor for OS (HR:10.240, 95%CI:2.161-48.512, *P* = 0.003) and PFS (HR:8.229, 95%CI:2.224-30.444, *P* = 0.002) (**Table 3**). This finding was confirmed by Kaplan-Meier survival analysis, as shown in **Figure 1**.

**Table 3:** Uni- and multivariate analyses for overall and progression-free survival in pN0 patients.

Variable	Overall survival		Progression-free survival	
	Hazard ratios (95%CI)	*P*	Hazard ratios (95%CI)	*P*
Univariate analyses				
Gender (Male *vs* Female)	0.746(0.201-2.769)	0.662	0.898(0.286-2.822)	0.854
Age (≤65 *vs*>65)	0.735(0.234-2.308)	0.598	0.792(0.316-2.408)	0.872
Smoker (No *vs* Yes)	1.847(0.581-5.871)	0.299	1.685(0.599-4.742)	0.323
Drinker (No *vs* Yes)	2.643(0.795-8.793)	0.113	2.109(0.670-6.633)	0.202
T-SUVmax (≤ 13.27 *vs >*13.27)	12.248(2.663-56.342)	0.001**	9.925(2.767-35.602)	<0.001***
T-MTV (≤ 17.54 *vs >*17.54)	5.802(1.682-20.010)	0.005**	4.967(1.684-14.649)	0.004**
T-TLG (≤ 71.09 *vs >*71.09)	5.184(1.131-23.772)	0.034*	3.962(1.117-14.048)	0.033*
L3-SMI (Low *vs* High)	0.022(0.000-2.901)	0.126	0.262(0.059-1.165)	0.079
LNY (≤ 10 *vs >*10)	0.307(0.091-1.029)	0.056	0.432(0.137-1.369)	0.154
pT stage (T1–2 *vs* T3)	1.546(0.487-4.906)	0.459	1.098(0.398-3.032)	0.857
DOI (≤ 5mm *vs >*5mm)	5.076(0.654-39.419)	0.120	2.876(0.648-12.764)	0.165
Margin (Negative *vs* Positive)	2.467(0.314-19.369)	0.390	1.867(0.245-14.251)	0.547
PNI (Negative *vs* Positive)	2.738(0.818-9.165)	0.102	2.751(0.938-8.072)	0.065
Adjuvant treatment (No *vs* Yes)	0.395(0.123-1.266)	0.118	0.557(0.201-1.545)	0.261
Multivariate analyses				
T-SUVmax (≤ 13.27 *vs >*13.27)	10.240(2.161-48.512)	0.003**	8.229(2.224-30.444)	0.002**

*P < 0.05, **P < 0.01, ***P < 0.001; CI, confidence intervals; T-SUVmax, tumor-maximum standardized uptake value; T-MTV, tumor- metabolic tumor volume; T-TLG, tumor-total lesion glycolysis; L3-SMI, L3 skeletal muscle index; LNY, lymph node yield; DOI, depth of invasion; PNI, perineural invasion. Bold values indicate P < 0.05.

### pN+ cohort

**Figure 2:**
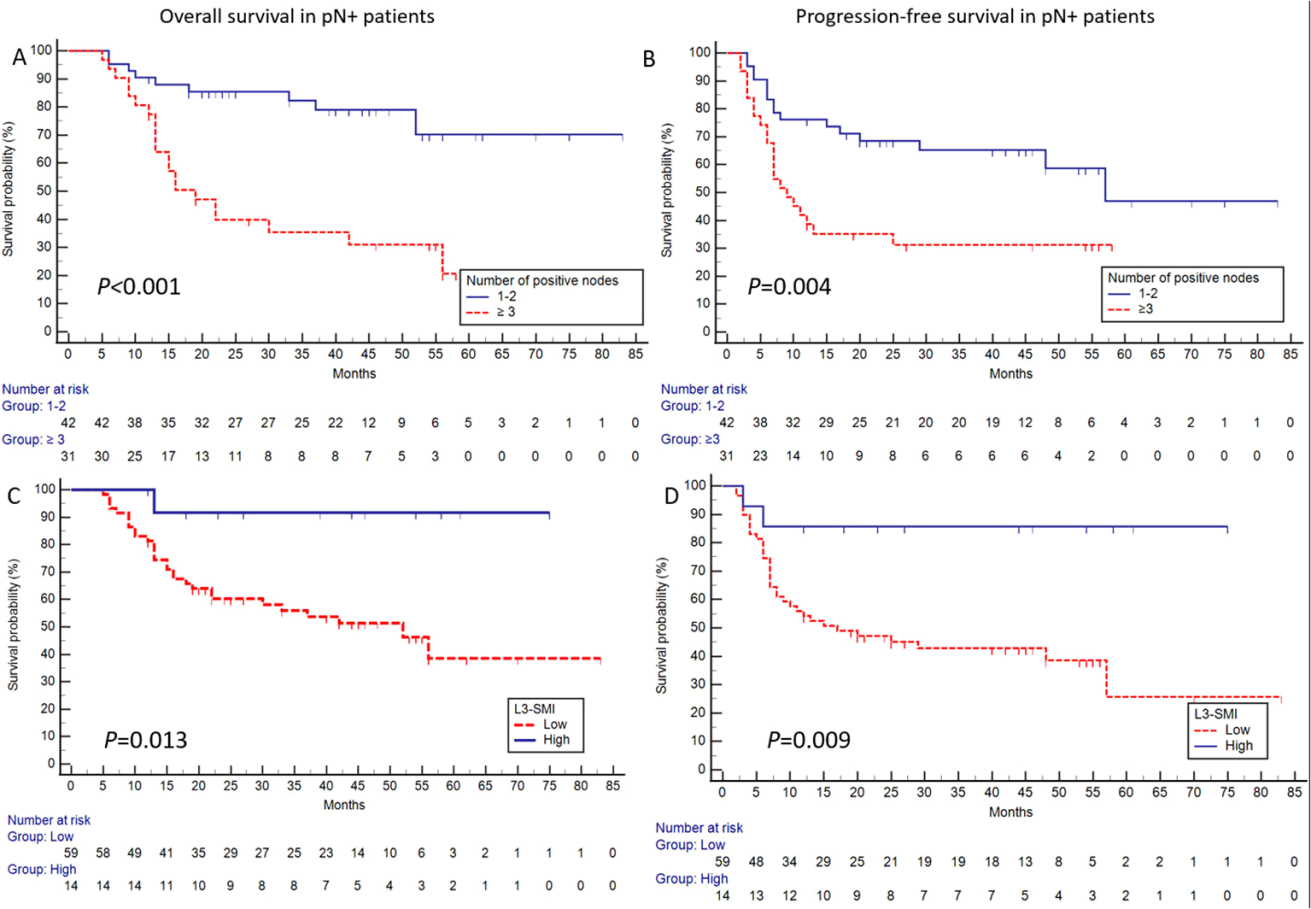
For pN+ patients, defined by the presence of ≥3 positive nodes, significant differences were observed in OS [*P* < 0.001; **(A)**] and PFS [*P* = 0.004; **(B)**] between the corresponding groups. Furthermore, patients with high L3-SMI exhibited superior OS [*P* = 0.013; **(C)**] and PFS [*P* = 0.009; **(D)**].

In pN+ patients, the univariate analysis for OS identified T-MTV, T-TLG, N-SUVmax, L3-SMI, the number of positive nodes, and LND as significant prognostic factors. Among these, the number of positive nodes (HR:4.151, 95%CI:1.887-9.133, *P* = 0.000) and L3-SMI (HR:0.133, 95%CI:0.018-0.975, *P* = 0.047) were independently associated with OS in the multivariate analysis. Similarly, for PFS, the univariate analysis identified T-MTV, N-SUVmax, L3-SMI, the number of positive nodes, and pT stage as significant prognostic factors. N-SUVmax (HR:2.343, 95%CI:1.151-4.769, *P* = 0.019), L3-SMI (HR:0.177, 95%CI:0.039-0.799, *P* = 0.024), pT stage (HR:3.739, 95%CI:1.367-10.230, *P* = 0.010), and the number of positive nodes (HR:1.971, 95%CI: 1.003-3.874, *P* = 0.049) emerge as independent predictors in the multivariate analysis (**Supplementary Table 2**). Notably, L3-SMI and the number of positive nodes were the two factors consistently associated with both OS and PFS, as confirmed by Kaplan-Meier survival analysis (**Figure 2**).

### Internal validation via bootstrap resampling

Bootstrap validation confirmed the prognostic value of key factors in both cohorts. In pN0 patients, T-SUVmax >13.27 was a relative stable predictor for both OS (median HR = 11.80, 95%CI: 3.38-41.95) and PFS (median HR = 10.36, 95%CI: 3.38-40.16). In pN+ patients, L3-SMI was a protective factor (median OS HR = 0.17, 95% CI: 0.07-0.51; median PFS HR = 0.20, 95%CI: 0.06-0.61), while a nodal burden of ≥3 nodes was a powerful risk factor (median OS HR = 4.36, 95%CI: 2.15-11.17; median PFS HR = 2.69, 95%CI: 1.41-5.56).

## Discussion

Our nodal-stratified analysis reveals distinct prognostic landscapes in OSCC: key metabolic and pathologic factors differ significantly between pN0 and pN+ patients (**Table 2**), indicating that nodal status not only stratifies but fundamentally reshapes risk determinants.

In OSCC patients with pN0 neck, Cox analysis identified T-SUVmax as the sole independent prognostic factor for both OS and PFS. Previous studies have reported its prognostic value, albeit with varying cutoff values [e.g., 7.2 by Lin et al. (3), 9.7 by Xu et al. (14), and 12 by Suzuki et al. (4)]. The prognostic significance of volumetric metabolic parameters like T-MTV has been inconsistent in the literature (5, 15), possibly due to two factors: analysis of combined pN0/pN+ cohorts and methodological variations in T-MTV measurement. Using a nodal-stratified design, our study established T-SUVmax as the primary metabolic prognosticator in pN0 cohort. Given the limited number of events in the pN0 cohort, the multivariable estimates, particularly the large hazard ratios with wide confidence intervals, should be interpreted with caution. However, the direction and magnitude of the association were supported by internal bootstrap validation.

In OSCC patients with pN+ neck, several studies have identified ENE (10), LNY (16), LND (or LNR) (16–18), and the number of positive nodes (18–22) as significant prognostic factors, while the role of L3-SMI remains underexplored. Our study, employing bootstrap validation, confirms that high nodal burden and sarcopenia are risk prognostic indicators for both OS and PFS in this cohort.

Our analysis identified a cutoff value of 3 for the number of positive nodes, consistent with the findings of Struckmeier et al. (18). The prognostic significance of this feature is further supported by Ho et al. (21), who proposed incorporating the number of positive nodes into N-stage modifications. Similarly, Roberts et al. (22) conducted a large-scale study involving 12,437 patients with head and neck squamous cell carcinoma (HNSCC). They concluded that the number of positive nodes outperformed LNR and AJCC N staging as a prognostic factor.

The prognostic role of L3-SMI in OSCC remains controversial. Some studies (7–9), using preoperative CT-derived L3-SMI, identified low SMI as an independent predictor of poor outcome in surgically treated OSCC patients. In contrast, other studies by Lu et al. (13) and Song et al. (23) found no significant association between sarcopenia and OSCC outcomes. Our study adds nuance to this controversy by suggesting that the prognostic impact of L3-SMI may be conditional on nodal status and treatment exposure. This association may be attributed to the dual role of low muscle mass in this setting: it may both reflect a poorer physiologic reserve, leading to relatively higher rates of adjuvant therapy incompletion (as observed in a subset of our pN+ cohort), and, among those who do undergo chemoradiotherapy, increase the risk of dose-limiting toxicity and poorer treatment outcomes as reported elsewhere (24, 25). Therefore, the prognostic significance of L3-SMI in pN+ disease appears to be mediated through its reflection of diminished physiologic reserve, which subsequently impacts treatment tolerance and completion. This suggests a treatment-related component to its prognostic value, rather than a purely biological one. Furthermore, the lack of a standardized sarcopenia threshold for the Chinese population complicates the interpretation of our results. Additional large-scale studies are needed to validate these findings and establish clinically relevant cutoff values.

This study further elucidates the prognostic value of N-SUVmax and pT stage in pN+ patients. Elevated N-SUVmax reflects heightened metabolic activity in metastatic lymph nodes, correlating with increased risk of neck recurrence, thereby supporting its role as an independent predictor of PFS (26). Concurrently, the association between pT stage and PFS in the pN+ cohort may stem from the higher prevalence of advanced pT stages within this subgroup (**Table 2**), validating its central role in traditional staging systems (2). Together, these factors complement the prognostic framework of positive lymph node count and L3-SMI.

This study has several limitations. First, the retrospective, single-center design with a limited sample size may introduce selection bias, which is evidenced by the wide confidence intervals for some estimates. Second, the low incidence of features like LVI and positive margins precluded a robust assessment of their prognostic role (27). Third, we acknowledge slight PET/CT-to-surgery timing variability and potential minor survival analysis noise. Fourth, the precision of depth of invasion (DOI) measurements was constrained by routine clinical practice. Finally, the analysis did not incorporate treatment variables such as chemotherapy dose or radiation field design, which may affect prognosis.

## Conclusions

In summary, our study suggests that a nodal status-stratified approach may improve risk assessment in OSCC. For pN0 disease, elevated T-SUVmax appears most relevant; for pN+ disease, high nodal burden combined with low muscle mass (sarcopenia) identifies patients at increased risk. This model, based on readily available imaging and pathology data, provides a preliminary framework for risk stratification. However, its clinical utility for guiding adjuvant therapy and surveillance must be prospectively validated.

## Study subjects or cohorts overlap

A subset of study subjects (78 out of 147) were included in a previously published paper (Xu F, et al. *Cancer Imaging* 2023). That prior study developed a prediction model for nodal metastasis in cN0 OSCC using metabolic and pathological variables. In contrast, the present study evaluates the prognostic value of metabolic variables, skeletal muscle index, and pathological features in OSCC patients with both pN0 and pN+ neck. The methodologies of the two studies are distinct and do not overlap.

## Data Availability

The original contributions presented in the study are included in the article/**Supplementary Material**. Further inquiries can be directed to the corresponding authors.
