# Supplementary material for: Nodal status dictates divergent prognostic drivers in oral squamous cell carcinoma: metabolic burden in pN0 vs. sarcopenia and nodal burden in pN+
Source: Front Oncol. 2026 Feb 18;16:1746241. doi: 10.3389/fonc.2026.1746241 (PMC12956693; doi:10.3389/fonc.2026.1746241)
Supplement: Supplementary file 1 [file Table1.docx]

Supplementary Table 1 Cutoff values for key variables using OS as the endpoint

| Variable | Cutoff value | Sensitivity (%) | Specificity (%) | AUC (95%CI) | *P* |
| --- | --- | --- | --- | --- | --- |
| pN0 patients |  |  |  |  |  |
| T-SUVmax | 13.27 | 83.3 | 69.4 | 0.759(0.645-0.851) | 0.001^**^ |
| T-MTV | 17.54 | 33.3 | 91.3 | 0.654(0.534-0.761) | 0.083 |
| T-TLG | 71.09 | 81.8 | 54.8 | 0.691(0.572-0.794) | 0.027^*^ |
| LNY | 10 | 33.3 | 88.7 | 0.544(0.424-0.660) | 0.655 |
| pN+ patients |  |  |  |  |  |
| T-SUVmax | 12.58 | 76.7 | 41.9 | 0.570(0.449-0.686) | 0.313 |
| T-MTV | 19.20 | 36.7 | 95.3 | 0.556(0.435-0.672) | 0.466 |
| T-TLG | 128.46 | 43.3 | 86.0 | 0.579(0.457-0.693) | 0.281 |
| N-SUVmax | 9.00 | 40.0 | 86.0 | 0.570(0.449-0.685) | 0.337 |
| LNY | 41 | 33.3 | 83.7 | 0.598(0.477-0.711) | 0.151 |
| Number of positive nodes | 3 | 70.0 | 74.4 | 0.776(0.664-0.866) | <0.001^***^ |
| LND | 0.0755 | 60.0 | 72.1 | 0.682(0.563-0.786) | 0.006^**^ |

**P*<0.05, ***P*<0.01, ****P*<0.001; CI: confidence intervals; T-SUVmax: tumor maximum standardized uptake value; T-MTV: tumor metabolic tumor volume; T-TLG: tumor total lesion glycolysis; N-SUVmax: node maximum standardized uptake value; LNY: lymph node yield; LND: lymph node density.
