# Supplementary material for: Nodal status dictates divergent prognostic drivers in oral squamous cell carcinoma: metabolic burden in pN0 vs. sarcopenia and nodal burden in pN+
Source: Front Oncol. 2026 Feb 18;16:1746241. doi: 10.3389/fonc.2026.1746241 (PMC12956693; doi:10.3389/fonc.2026.1746241)
Supplement: Supplementary file 2 [file Table2.docx]

Supplementary Table 2.

Uni- and multivariate analyses for overall and progression-free survival in pN+ patients

| **Variable** | **Overall survival** | | **Progression-free survival** | |
| --- | --- | --- | --- | --- |
|  | **Hazard ratios(95%CI)** | ***P*** | **Hazard ratios(95%CI)** | ***P*** |
| **Univariate analyses** |  |  |  |  |
| Gender Male *vs* Female | 0.520(0.181-1.491) | 0.223 | 0.545(0.212-1.400) | 0.207 |
| Age (≤65*vs* >65) | 0.858(0.392-1.875) | 0.701 | 0.697(0.337-1.442) | 0.330 |
| Smoker (No *vs* Yes) | 0.671(0.305-1.477) | 0.322 | 0.776(0.385-1.564) | 0.478 |
| Drinker (No *vs* Yes) | 0.861(0.383-1.939) | 0.718 | 0.893(0.429-1.861) | 0.764 |
| T-SUVmax (≤ 12.58 *vs* >12.58) | 1.916(0.821-4.471) | 0.132 | 1.925(0.907-4.085) | 0.088 |
| 1. MTV (≤19.2 *vs* >19.2) | 3.248(1.538-6.860) | **0.002**** | 2.746(1.349-5.591) | **0.005**** |
| T-TLG (≤128.46 *vs* >128.46) | 2.446(1.184-5.051) | **0.016*** | 1.856(0.942-3.660) | 0.074 |
| 1. SUVmax (≤9 *vs* >9) | 2.474(1.186-5.160) | **0.016*** | 2.209(1.121-4.353) | **0.022*** |
| L3-SMI (Low *vs* High) | 0.123(0.017-0.907) | **0.040*** | 0.187(0.045-0.781) | **0.022*** |
| DOI (≤ 5mm *vs* >5mm) | 4.467(0.605-32.996) | 0.142 | 3.041(0.720-12.852) | 0.130 |
| PNI (Negative *vs* Positive) | 1.822(0.884-3.752) | 0.104 | 1.720(0.896-3.303) | 0.103 |
| LVI (Negative *vs* Positive) | 2.949(0.694-12.532) | 0.143 | 2.592(0.613-10.957) | 0.195 |
| Margin (Negative *vs* Positive) | 0.044(0.000-37.344) | 0.363 | 0.044(0.000-18.321) | 0.311 |
| Grade (Moderate *vs* Poor) | 0.717(0.217-2.371) | 0.585 | 0.981(0.346-2.779) | 0.971 |
| ENE (Negative *vs* Positive) | 1.394(0.677-2.874) | 0.368 | 1.547(0.807-2.965) | 0.189 |
| LNY (≤41 *vs* >41) | 1.344(0.627-2.881) | 0.448 | 1.792(0.837-3.838) | 0.133 |
| Number of positive nodes (1-2 *vs* ≥3) | 4.840(2.197-10.664) | **<0.001***** | 2.744(1.419-5.305) | **0.003**** |
| LND (≤0.0755 *vs* >0.0755) | 2.762(1.310-5.825) | **0.008**** | 1.714(0.898-3.269) | 0.102 |
| pT stage (T1-2 *vs* T3-4) | 2.491(0.867-7.153) | 0.090 | 2.908(1.130-7.485) | **0.027*** |
| pN stage (N1-2 *vs* N3) | 2.037(0.778-5.334) | 0.147 | 1.566(0.715-3.428) | 0.262 |
| Adjuvant treatment (No *vs* Yes) | 0.599(0.181-1.987) | 0.402 | 0.234(0.234-2.498) | 0.657 |
| **Multivariate analyses** |  |  |  |  |
| Number of positive nodes (1-2 *vs* ≥3) | 4.151(1.887-9.133) | **<0.001***** | 1.971(1.003-3.874) | **0.049*** |
| L3-SMI (Low *vs* High) | 0.133(0.018-0.975) | **0.047*** | 0.177(0.039-0.799) | **0.024*** |
| N-SUVmax (≤9 *vs* >9) |  |  | 2.343(1.151-4.769) | **0.019*** |
| pT stage (T1-2 *vs* T3-4) |  |  | 3.739(1.367-10.230) | **0.010*** |

**P*<0.05, ***P*<0.01, ****P*<0.001; CI: confidence intervals; T-SUVmax: tumor maximum standardized uptake value; T-MTV: tumor metabolic tumor volume; T-TLG: tumor total lesion glycolysis; N-SUVmax: node maximum standardized uptake value; L3-SMI: L3 skeletal muscle index; DOI: depth of invasion; PNI: perineural invasion; LVI: Lymphovascular invasion; ENE: extracapsular invasion; LNY: lymph node yield; LND: lymph node density.
